# Detection of familial hypercholesterolaemia: external validation of the FAMCAT clinical case-finding algorithm to identify patients in primary care

**DOI:** 10.1016/S2468-2667(19)30061-1

**Published:** 2019-05-01

**Authors:** Stephen Weng, Joe Kai, Ralph Akyea, Nadeem Qureshi

**Affiliations:** a Primary Care Stratified Medicine (PRISM), Division of Primary Care, University of Nottingham, Nottingham, UK

## Abstract

**Background:**

The vast majority of individuals with familial hypercholesterolaemia in the general population remain unidentified worldwide. Recognising patients most likely to have the condition, to enable targeted specialist assessment and treatment, could prevent major coronary morbidity and mortality. We aimed to evaluate a clinical case-finding algorithm, the familial hypercholesterolaemia case ascertainment tool (FAMCAT), and compare it with currently recommended methods for detection of familial hypercholesterolaemia in primary care.

**Methods:**

In this external validation study, FAMCAT regression equations were applied to a retrospective cohort of patients aged 16 years or older with cholesterol assessed, who were randomly selected from 1500 primary care practices across the UK contributing to the QResearch database. In the main analysis, we assessed the ability of FAMCAT to detect familial hypercholesterolaemia (ie, its discrimination) and compared it with that of other established clinical case-finding approaches recommended internationally (Simon Broome, Dutch Lipid Clinic Network, Make Early Diagnosis to Prevent Early Deaths [MEDPED] and cholesterol concentrations higher than the 99th percentile of the general population in the UK). We assessed discrimination by area under the receiver operating curve (AUROC; ranging from 0·5, indicating pure chance, to 1, indicating perfect discrimination). Using a probability threshold of more than 1 in 500 (prevalence of familial hypercholesterolaemia), we also assessed sensitivity, specificity, positive predictive values, and negative predictive values in the main analysis.

**Findings:**

A sample of 750 000 patients who registered in 1500 UK primary care practices that contribute anonymised data to the QResearch database between Jan 1, 1999, and Sept 1, 2017, was randomly selected, of which 747 000 patients were assessed. FAMCAT showed a high degree of discrimination (AUROC 0·832, 95% CI 0·820–0·845), which was higher than that of Simon Broome criteria (0·694, 0·681–0·703), Dutch Lipid Clinic Network criteria (0·724, 0·710–0·738), MEDPED criteria (0·624, 0·609–0·638), and screening cholesterol concentrations higher than the 99th percentile (0·581, 0·570–0·591). Using a 1 in 500 probability threshold, FAMCAT achieved a sensitivity of 84% (1028 predicted *vs* 1219 observed cases) and specificity of 60% (443 949 predicted *vs* 745 781 observed non-cases), with a corresponding positive predictive value of 0·84% and a negative predictive value of 99·2%.

**Interpretation:**

FAMCAT identifies familial hypercholesterolaemia with greater accuracy than currently recommended approaches and could be considered for clinical case finding of patients with the highest likelihood of having hypercholesterolaemia in primary care.

**Funding:**

UK National Institute for Health Research School for Primary Care Research.

## Introduction

Familial hypercholesterolaemia is the commonest inherited autosomal dominant disorder and causes elevated serum LDL cholesterol from birth.^1^ It affects between 1 in 200 and 1 in 500 individuals in the general population,^2^, ^3^ but the vast majority of cases are unrecognised worldwide.^4^ In the UK, for example, more than 80% of an estimated 320 000 individuals remain undiagnosed, resulting in major lost opportunities to prevent premature heart disease and death.^5^ If it is left untreated, premature coronary heart disease will develop in approximately 50% of men with familial hypercholesterolaemia by the age of 50 years and about 30% of women with familial hypercholesterolaemia by the age of 60 years.^6^ Individuals with untreated familial hypercholesterolaemia have a 100-fold increase in coronary heart disease mortality risk compared with the general population.^7^, ^8^ Such risk can be very effectively prevented with high-potency lipid-lowering treatment, which can halve coronary heart disease mortality.^9^

Although clinical cardiovascular risk assessment routinely includes serum cholesterol measurement, it fails to effectively identify people at increased risk of familial hypercholesterolaemia.^5^ Therefore, internationally, case finding for familial hypercholesterolaemia is recommended using recognised specialist criteria, such as the Dutch Lipid Clinic Network (DLCN), Simon Broome, or Make Early Diagnosis to Prevent Early Deaths (MEDPED) criteria.^10^, ^11^, ^12^ In addition to these criteria, UK guidelines^10^ suggest that a cholesterol concentration of more than 9 mmol/L in individuals older than 30 years and more than 7·5 mmol/L for those aged 30 years or younger (in line with the 99th percentile for the general population) as a starting point for familial hypercholesterolaemia case finding. The Simon Broome critieria,^10^ most commonly used in the UK, recommend that individuals with a total cholesterol concentration of more than 7·5 mmol/L and a family history of premature heart disease should be classified as having probable familial hypercholesterolaemia in primary care and should be referred for further lipid specialist assessment. Patients who then also meet specific clinical diagnostic criteria (eg, tendon xanthoma), or diagnosis by genetic testing, are categorised as having definite familial hypercholesterolaemia. The DLCN criteria^11^ use a points-based scoring system to classify possible, probable, or definite familial hypercholesterolaemia on the basis of differing LDL cholesterol thresholds, family history of premature vascular disease and raised cholesterol, personal history of premature vascular disease, clinical signs such as tendon xanthoma and arcus senilis, or mutation status. The MEDPED criteria^12^ use age-stratified total cholesterol thresholds for both the general population and relatives depending on degree of relation.

**Research in context**

**Evidence before this study**

Familial hypercholesterolaemia is one of the commonest inherited disorders in the general population, with 50% of people affected developing premature heart disease by 50 years of age. More than 80% of an estimated 320 000 individuals in the UK remain undiagnosed, resulting in lost opportunities to effectively prevent premature heart disease. We searched MEDLINE and Embase for research articles published in English between Jan 1, 1946, and Sept 15, 2018, using search terms: “familial hypercholesterol*”, “diagnos*”, “identif*”, “case finding”, “risk algorithm”, “Simon Broom*”, “Dutch Lipid*”, “MEDPED”, “FAMCAT”, and “primary care”. We identified that most previous studies have used the Simon Broome or Dutch Lipid Clinic Network criteria for identification of familial hypercholesterolaemia in primary care. Familial hypercholesterolaemia case ascertainment tool (FAMCAT) was the only case-finding algorithm to identify familial hypercholesterolaemia that has been developed from a large primary care database.

**Added value of this study**

This study has now externally validated FAMCAT using a separate cohort of 747 000 patients from 1500 primary medical care practices in the UK, from a different and the world's largest primary care database. FAMCAT is confirmed to have high predictive accuracy to detect patients at highest likelihood of having familial hypercholesterolaemia in the general primary care population and performs better than use of Simon Broome, Make Early Diagnosis to Prevent Early Deaths (MEDPED), Dutch Lipid Clinic Network criteria, and very high cholesterol concentrations alone.

**Implications of all the available evidence**

The current study underlines the poor predictive accuracy of simply using very elevated cholesterol concentrations alone to identify familial hypercholesterolaemia, and that specialist Simon Broome, MEDPED, and Dutch Lipid Clinical Network criteria have relatively lower accuracy than FAMCAT when applied to primary care. For clinical case finding of patients in primary care with highest likelihood of having familial hypercholesterolaemia, FAMCAT is more accurate than other approaches and could be used to improve detection of familial hypercholesterolaemia in the general population.

The routine application of DLCN, Simon Broome, or MEDPED criteria to patients in generalist clinical practice, such as primary care, is a practical challenge given the assessments required, including collection of detailed family history. Moreover, as many as one in four patients in the general population with cholesterol concentrations of more than 7·5 mmol/L fulfil referral criteria for probable familial hypercholesterolaemia, most of whom will not transpire to have definite familial hypercholesterolaemia when further assessed by lipid specialists^13^ or genetic diagnosis.^14^ A simpler and more effective clinical case-finding approach to identify patients with the highest risk of having familial hypercholesterolaemia is needed. Such an approach could improve targeted referral for lipid specialist assessment and diagnosis, while reducing unnecessary referrals, to better detect the vast majority of individuals with familial hypercholesterolaemia in the general population who remain undiagnosed.

A clinical case-finding algorithm, familial hypercholesterolaemia case ascertainment tool (FAMCAT), has recently been derived and validated using data from almost 3 million primary care patients, including more than 5000 cases of familial hypercholesterolaemia, from the Clinical Practice Research Datalink (CPRD) of 681 primary care practices in the UK.^15^ The algorithm had high predictive accuracy to identify primary care patients with the greatest probability of having familial hypercholesterolaemia, with an area under the receiver operating curve (AUROC) of 0·86.^15^ It has begun to be integrated in primary care computer systems as a case-finding tool.^16^

The majority of clinical algorithms lack well conducted and clearly reported external validation.^17^ We aimed to provide external validation of the FAMCAT clinical case-finding algorithm to identify patients in primary care at the highest risk of having familial hypercholesterolaemia. We used a separate clinical database, from 1500 UK primary care practices, which had no overlap with the database from which FAMCAT was derived.

## Methods

### Study design and population

We did a retrospective cohort study in a large population of primary care patients, using the QResearch database. We randomly selected, using a random number generator, a sample of adult patients aged 16 years or older, who registered in 1500 UK primary care practices that contribute anonymised data to the QResearch database and had at least one documented total or LDL cholesterol measurement (necessary for establishing a suspected diagnosis). The cohort comprised all patients who were actively registered and contributing data (had visited their practice up until the end date of when data were extracted). For patients who were identified with familial hypercholesterolaemia, the date of diagnosis was specified as their ending date to ensure all predictors remained temporal to their familial hypercholesterolaemia outcome.

Patients aged younger than 16 years were excluded from the analysis because cholesterol thresholds for diagnosis and treatment of familial hypercholesterolaemia in children differ from those for adults.^10^ Patients were also excluded if they had a previous familial hypercholesterolaemia diagnosis before the study entry date or a diagnosis of other inherited lipid disorders.

The selected baseline registration date corresponded to the same starting time used when deriving the FAMCAT algorithm using CPRD.^15^ Moreover, the 750 000 randomly selected patient sample was similar to the size of that for previous FAMCAT internal validation done in CPRD using 742 851 patients, providing a comparable sample for external validation.

### Predictor variables

FAMCAT was developed as a multivariable logistic regression model, stratified by sex, to calculate an individual's probability of having familial hypercholesterolaemia. The panel summarises all ten predictors that were incorporated into FAMCAT. The variables were extracted from the QResearch database using UK National Health Service (NHS) read code lists (appendix pp 6–23) during the study period. Age, cholesterol concentrations, and triglycerides were categorised (panel). Statin potency was determined using classifications in the most recent UK National Institute for Health and Care Excellence (NICE) lipid modification guidelines.^18^ Secondary causes of raised cholesterol, such as diabetes and chronic kidney disease, were included as predictor variables for lower probability of familial hypercholesterolaemia. Full details of FAMCAT development, internal validation, variable definitions, effect sizes, and prediction performance are published elsewhere.^15^

Panel **Summary of predictor variables in FAMCAT**

• Sex (male or female)
• Age in years (16–24, 25–34, 35–44, 45–54, 55–64, 65–74, or 75–84)
• Highest cholesterol measurement recorded
  • Ideal: total cholesterol ≤5 mmol/L or LDL cholesterol ≤3·3 mmol/L
  • High: total cholesterol 5·1–6·5 mmol/L or LDL cholesterol 3·4–4·1 mmol/L
  • Very high: total cholesterol 6·6–7·5 mmol/L or LDL cholesterol 4·2–4·9 mmol/L
  • Extremely high: total cholesterol >7·5 mmol/L or LDL cholesterol >4·9 mmol/L
• Triglycerides within 1 month of highest cholesterol measurement (mmol/L)
  • Ideal: <1·7 mmol/L
  • Borderline high: 1·7–2·2 mmol/L
  • High: 2·3–5·5 mmol/L
  • Very high: ≥5·6 mmol/L
• Lipid-lowering drugs prescribed within 1 month of highest cholesterol measurement (none, fibrate, bile acid sequestrant, nicotinic acid, low-potency statin, medium-potency statin, or high-potency statin)
• Family history of familial hypercholesterolaemia (no or yes)
• Family history of myocardial infarction (no or yes)
• Family history of raised cholesterol (no or yes)
• Type 1 or type 2 diabetes (no or yes)
• Chronic kidney disease (no or yes)

### Outcomes

The primary outcome was defined as the incident diagnosis of familial hypercholesterolaemia, identified from a patient record. In the UK, diagnosis of familial hypercholesterolaemia is made by lipid specialists following clinical assessment using specific diagnostic criteria or genetic testing.^10^ Familial hypercholesterolaemia is then specifically coded in the UK primary care coding system by NHS read codes, which are a coded hierarchy of clinical terms that provide a standard way by which clinicians can record patients' findings (grouped in families of codes) and procedures in information technology systems across primary and secondary care. In the hypercholesterolaemia family of read codes, the familial hypercholesterolaemia code is a specific subcode.

### Statistical analysis

Assuming a population frequency of familial hypercholesterolaemia between 1 in 500 (0·002) and 1 in 250 (0·004), to achieve an AUROC of at least 0·80, with 90% power and 5% significance requires between 46 690 (190 positive, 46 500 negative) and 95 190 samples (190 positive, 95 000 negative).

We applied the FAMCAT logistic regression equations directly to every patient in the cohort to calculate each patient's probability of having familial hypercholesterolaemia by use of the untransformed regression coefficients and constant terms provided in the appendix p 1. We provided descriptive characteristics of the study population as patient demographics and clinical characteristics. We described continuous normally distributed variables by mean and SD, and continuous non-normally distributed variables by median and IQR. We presented categorical variables as number and proportions. We used multiple imputation with chained equations to estimate missing values for triglyceride measurements (which were sometimes not assessed with cholesterol measurements) by generating ten imputed datasets.^19^

We assessed prediction accuracy by examining measures of calibration and discrimination. Calibration was defined as how closely the predicted probability of familial hypercholesterolaemia agrees with the expected probability of familial hypercholesterolaemia. We assessed calibration by plotting the observed number of cases of familial hypercholesterolaemia against the predicted number of cases of familial hypercholesterolaemia for each tenth of predicted probability to ensure ten equally sized groups (deciles).

Discrimination was defined as the ability of the algorithm to differentiate between patients who were predicted to have familial hypercholesterolaemia and those who do not have familial hypercholesterolaemia. This measure was quantified by calculating the AUROC, which is equal to the *c* statistic (concordance). The AUROC value gave the probability that a randomly selected patient who has familial hypercholesterolaemia has a higher probability score than a patient who does not have familial hypercholesterolaemia, ranging from 0·5 (pure chance) to 1 (perfect discrimination). To generate 95% CIs for the AUROC, we used a jack-knife procedure^20^ to estimate SEs.

In the primary analysis, we also compared the discrimination of FAMCAT against Simon Broome,^10^ DLCN,^11^ and MEDPED^12^ diagnostic criteria, and a simple classification of total cholesterol concentration higher than the 99th percentile^10^ for determining possible or probable familial hypercholesterolaemia. Predictors included in the Simon Broome and DLCN criteria were extracted using NHS read codes and applied directly to the cohort. In a subsequent subgroup analysis, we assessed discrimination across various ethnic groups.

As the predicted probabilities of familial hypercholesterolaemia varied across a continuum, case finding for familial hypercholesterolaemia in primary care requires decision makers to set a probability threshold. The FAMCAT algorithm was shown in an internal validation analysis^15^ to have 70% sensitivity and 88% specificity when using a probability threshold of more than 1 in 500 to determine familial hypercholesterolaemia. This threshold corresponds to a conservatively estimated frequency of familial hypercholesterolaemia (based on the Hardy-Weinberg equation).^2^ Using the same threshold of more than 1 in 500, we calculated the sensitivity, specificity, positive predictive value, and negative predictive value.

In a post-hoc analysis, we also calculated the sensitivity, specificity, positive predictive value, and negative predictive assuming a probability threshold of greater 1 in 250 to reflect the more recent prevalence figures for familial hypercholesterolaemia in the general population.^3^

In a sensitivity analysis to further enhance the accuracy of the FAMCAT algorithm, we assessed the benefit of using lipid measurements (total cholesterol, LDL cholesterol, and triglycerides) and age as continuous variables, with an interaction term to specify whether the measurement was either total cholesterol or LDL cholesterol. Normality was assessed by the Kolmogorov-Smirnov test and, if found to be significant, the variables were log-transformed before inclusion in the multivariable model. Personal history of premature myocardial infarction (as this features in the DLCN criteria), which the primary FAMCAT algorithm does not include, was also included in the model. Premature myocardial infarction was defined as having an event before the age of 55 years for men and before the age of 60 years for women.

We used MedCalc software, version 18.11.6, to estimate the sample size.

### Role of the funding source

The funder of the study had no role in study design, data collection, data analysis, data interpretation, or writing of the report. The corresponding author had full access to all of the data and the final responsibility for the decision to submit for publication.

## Results

Of the 750 000 patients who registered at participating primary care facilities between Jan 1, 1999, and Sept 1, 2017, 3000 patients were excluded because of having other inherited lipid disorders or having all of their cholesterol measurements after a diagnosis of familial hypercholesterolaemia. There were 1219 cases (0·2%) of familial hypercholesterolaemia in the study cohort (table 1).

**Table 1:** Characteristics of patients aged 16 years from the validation cohort

		**Men (n=362 769)**	**Women (n=384 231)**
Familial hypercholesterolaemia diagnosis		485 (0·1%)	734 (0·2%)
Age, years		51·2 (15·7)	52·4 (17·3)
Age during cholesterol measurement, years		54·6 (15·3)	56·5 (16·8)
History of premature myocardial infarction*		12 712 (3·5%)	4333 (1·1%)
Ethnicity			
	White, white British, or other white	189 239 (52·2%)	210 914 (54·9%)
	Asian, Asian British, or other Asian	5361 (1·5%)	6011 (1·6%)
	Black, black British, African, or Caribbean	9231 (2·5%)	11 204 (2·9%)
	Mixed or multiple ethnic groups	17 454 (4·8%)	16 847 (4·4%)
	Other ethnic group	12 137 (3·3%)	13 320 (3·5%)
	Unknown or not recorded	129 347 (35·7%)	125 935 (32·8%)
Lipid profile			
	Highest total cholesterol recorded, mmol/L	5·6 (1·2)	5·8 (1·3)
	Highest LDL cholesterol recorded, mmol/L	3·2 (1·1)	3·2 (1·0)
	Triglycerides during cholesterol measurement, mmol/L†	2·2 (1·7)	1·7 (1·2)
Lipid-lowering drug usage at time of cholesterol measurement			
	Prescribed fibrate, bile acid sequestrant, or nicotinic acid	885 (0·2%)	731 (0·2%)
	Prescribed low-potency statin	4739 (1·3%)	3658 (1·0%)
	Prescribed medium-potency statin	16 669 (4·6%)	13 431 (3·5%)
	Prescribed high-potency statin	5597 (1·5%)	3848 (1·0%)
Family history			
	Family history of familial hypercholesterolaemia	949 (0·3%)	1526 (0·4%)
	Family history of raised cholesterol	2554 (0·7%)	3718 (1·0%)
	Family history of myocardial infarction	11 300 (3·1%)	13 499 (3·5%)
Secondary causes of high cholesterol at time of cholesterol measurement			
	Diabetes diagnosis	54 680 (15·1%)	44 834 (11·7%)
	Chronic kidney disease diagnosis	32 551 (9·0%)	43 911 (11·4%)

Data are n (%) or mean (SD).

* Premature is defined as younger than 55 years in men and younger than 60 years in women.

† Data missing for 116 532 (15·6%) of 747 000 participants.

The mean highest total cholesterol was slightly higher in women (5·8 mmol/L [SD 1·3]) than in men (5·6 mmol/L [1·2]). The mean highest LDL cholesterol (3·2 mmol/L [SD 1·1] in men and 3·2 mmol/L [1·0] in women) was the same for both sexes. The majority of the sample were of white ethnicities (189 239 [52·2%] of 362 769 men and 210 914 [54·9%] of 384 231 women). 11 300 (3·1%) of 362 769 men and 13 499 (3·5%) of 384 231 women had a family history of myocardial infarction. Secondary causes of hypercholesterolaemia such as diabetes (54 680 [15·1%] of 362 769 men and 44 834 [11·7%] of 384 231 women) and chronic kidney disease (32 551 [9·0%] of 362 769 men and 43 911 [11·4%] of 384 231 women) were common.

External validation of the FAMCAT model in QResearch showed a high level of discrimination (AUROC 0·832, 95% CI 0·820–0·845; table 2). FAMCAT showed significantly better discrimination than Simon-Broome criteria (AUROC 0·694, 95% CI 0·681–0·703), DLCN criteria (0·724, 0·710–0·738), and MEDPED criteria (0·624, 0·609–0·638). The new NICE recommendation of screening for cholesterol concentration higher than the 99th percentile showed poor discrimination (AUROC 0·581, 95% CI 0·570–0·591). See the appendix p 2 for AUROCs stratified by sex.

**Table 2:** Model discrimination in the external validation cohort for identification of familial hypercholesterolaemia in primary care (n=747 000)

	**AUROC**	SE*	**95% CI**
FAMCAT	0·832	0·006	0·820–0·845
Simon Broome criteria†	0·694	0·007	0·681–0·703
Dutch Lipid Clinic Network criteria‡	0·724	0·007	0·710–0·738
MEDPED criteria§	0·624	0·007	0·609–0·638
Cholesterol concentration higher than the 99th percentile¶	0·581	0·005	0·570–0·591

AUROC=area under the receiver operating curve. FAMCAT=familial hypercholesterolaemia case ascertainment tool. MEDPED=Make Early Diagnosis to Prevent Early Deaths.

* Jack-knife procedure to estimate SEs.^20^

† Total cholesterol >7·5 mmol/L or LDL cholesterol >4·9 mmol/L and family history of premature myocardial infarction.^10^

‡ Score based on LDL cholesterol, family history, clinical history, and physical examination.^11^

§ Age-stratified total cholesterol thresholds for the general population.^12^

¶ Total cholesterol >9·0 mmol/L or LDL cholesterol >6·6 mmol/L if age was older than 30 years; total cholesterol >7·5 mmol/L or LDL cholesterol > 4·9 mmol/L if age was 30 years or younger.^10^

**Figure 1:**
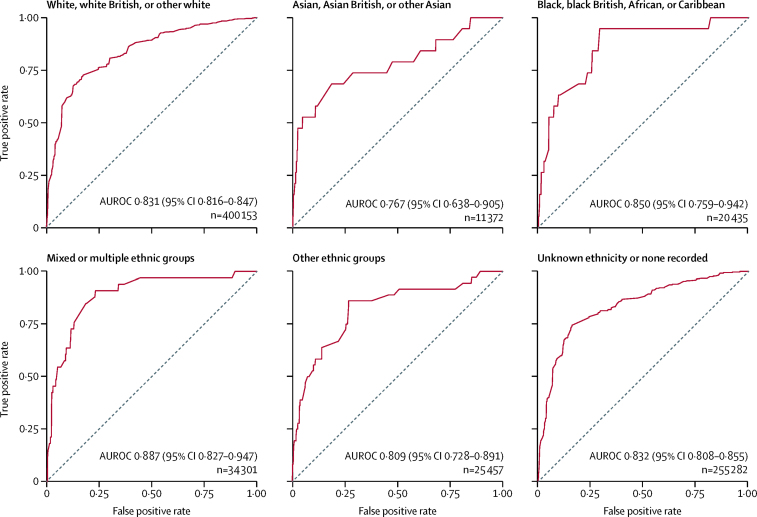
AUROCs derived from the external validation cohort (n=747 000) for FAMCAT subgroup analysis Higher area under the curve (*c* statistic) confers better discrimination. AUROC=area under the receiver operating curve. FAMCAT=familial hypercholesterolaemia case ascertainment tool.

The accuracy of FAMCAT for prediction of familial hypercholesterolaemia by ethnic group resulted in discrimination ranging from 0·767 (95% CI 0·638–0·905) for the Asian, Asian British, or other Asian group (n=11 372) to 0·887 (0·827–0·947) for the mixed or multiple ethnic group (n=34 301; figure 1). In the largest group represented—white, white British, or other white ethnic (n=400 153)—discrimination was identical to the overall discrimination of FAMCAT. There were also a large number of individuals (n=255 282) without any documented ethnicity in their electronic primary care records.

The model showed good calibration across most deciles between observed and predicted cases, with some under prediction of cases in the highest decile (table 3). There was an expected sharp increase in observed and predicted cases in the highest decile of predicted probability where 752 cases were observed and 638 cases were predicted.

**Table 3:** FAMCAT model calibration of observed versus predicted cases of familial hypercholesterolaemia in the external validation cohort by deciles of predicted probability

	**Probability of familial hypercholesterolaemia**	**Observed cases**	**Predicted cases**
1 (lowest decile)	0·0002	19	12
2	0·0003	18	17
3	0·0004	28	27
4	0·0006	25	36
5	0·0007	53	76
6	0·0010	51	31
7	0·0015	81	125
8	0·0016	53	114
9	0·0035	139	144
10 (highest decile)	0·1753	752	638

FAMCAT=familial hypercholesterolaemia case ascertainment tool.

In terms of case finding in primary care practice, we considered a threshold corresponding to the estimated frequency of familial hypercholesterolaemia in the general population between 1 in 500 and 1 in 250. Using a cutoff above 1 in 500 (0·002), FAMCAT achieved a sensitivity of 84% (1028 predicted cases *vs* 1219 observed cases) and specificity of 60% (443 949 predicted non-cases *vs* 745 781 observed non-cases), with a corresponding positive predictive value of 0·84% and a negative predictive value of 99·2%. Using a cutoff above 1 in 250 (0·004) in a post-hoc analysis, FAMCAT achieved a sensitivity of 72% (878 predicted cases *vs* 1219 observed cases) and specificity of 84% (624 349 predicted non-cases *vs* 745 781 observed non-cases), with a corresponding positive predictive value of 1·8% and a negative predictive value of 98·2%.

In the sensitivity analysis, cholesterol concentrations, being prescribed lipid-lowering drugs, history of premature myocardial infarction, and having a family history of myocardial infarction, familial hypercholesterolaemia, or raised cholesterol significantly increased likelihood that an individual has familial hypercholesterolaemia (table 4). Increasing triglyceride concentration and age, and presence of secondary causes of hypercholesterolaemia (diabetes or chronic kidney disease), decreased the likelihood that an individual has familial hypercholesterolaemia because these factors are more likely to indicate a non-inherited cause of raised cholesterol (table 4).

**Table 4:** Adjusted odds ratios from logistic regression model of FAMCAT with continuous values for all lipid measurements and age with inclusion of personal history of premature myocardial infarction

	**Men (n=362 769)**	**Women (n=384 231)**
**Highest cholesterol recorded, mmol/L**		
If LDL cholesterol measured	2·57 (2·43–2·73)	3·29 (3·13–3·47)
If total cholesterol measured	1·70 (1·61–1·80)	1·95 (1·83–2·07)
Age during cholesterol measurements, years	0·97 (0·96–0·98)	0·99 (0·98–1·00)
Log triglycerides during cholesterol measurement, mmol/L	0·16 (0·13–0·19)	0·08 (0·06–0·09)
Lipid-lowering drugs prescribed during cholesterol measurement*		
Prescribed fibrate, bile acid sequestrant, or nicotinic acid	7·03 (2·42–20·36)	3·98 (1·20–13·28)
Prescribed low-potency statin†	1·03 (0·25–4·18)	3·54 (1·72–7·30)
Prescribed medium-potency statin‡	1·64 (1·29–2·41)	1·72 (1·03–2·87)
Prescribed high-potency statin§	1·75 (0·79–3·91)	2·55 (1·32–4·91)
Previously history of premature myocardial infarction*‖	2·30 (1·49–3·53)	1·54 (0·79–3·00)
Family history of familial hypercholesterolaemia*	6·74 (3·98–11·39)	2·49 (1·46–2·25)
Family history of myocardial infarction*	3·53 (2·24–5·57)	4·37 (3·17–6·04)
Family history of raised cholesterol*	2·80 (1·69–4·64)	2·12 (1·40–3·22)
Diabetes diagnosis*	0·25 (0·13–0·46)	0·46 (0·29–0·74)
Chronic kidney disease diagnosis*	0·77 (0·46–1·32)	0·17 (0·10–0·30)

Data are adjusted odds ratios (95% CI). Full regression coefficients are presented in the appendix p 3. FAMCAT=familial hypercholesterolaemia case ascertainment tool.

* Compared with reference group (odds ratio 1).

† Fluvastatin or pravastatin 40 mg per day, or simvastatin 10 mg per day.

‡ Fluvastatin or pravastatin 80 mg per day, simvastatin 20–40 mg per day, atorvastatin 10 mg per day, or rosuvastatin 5 mg per day.

§ Simvastatin 80 mg per day, atorvastatin 20 mg per day, or rosuvastatin 10 mg per day.

‖ Premature defined as younger than 55 years in men and younger than 60 years in women.

**Figure 2:**
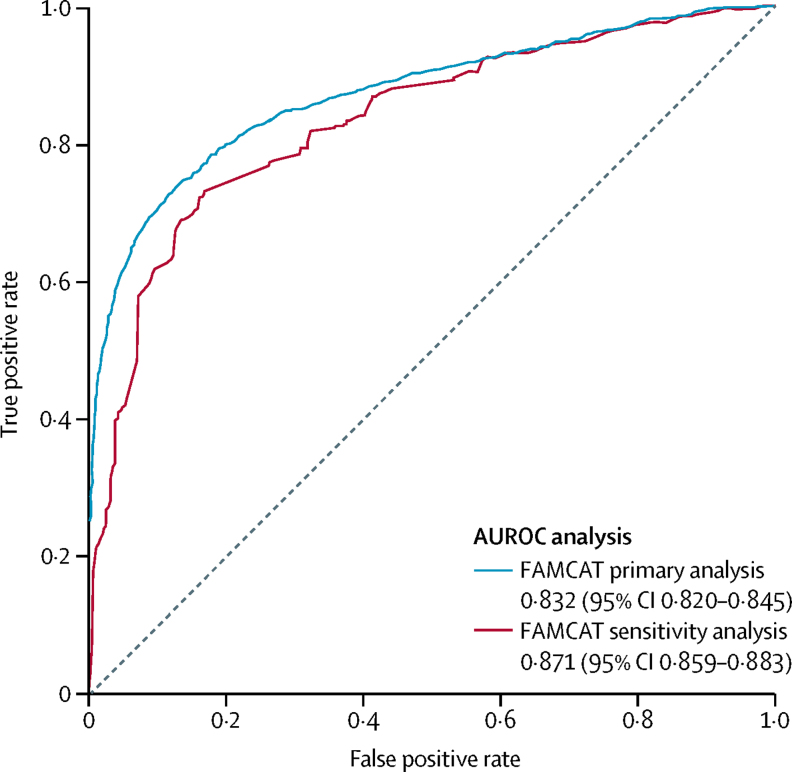
AUROCs derived from the external validation cohort (n=747 000) for FAMCAT sensitivity analysis Higher area under the curve (*c* statistic) confers better discrimination. AUROC=area under the receiver operating curve. FAMCAT=familial hypercholesterolaemia case ascertainment tool.

In terms of discrimination, this sensitivity analysis model also further enhanced accuracy of overall performance by 4% compared with FAMCAT assessed in the primary analysis, with an AUROC of 0·871 (95% CI 0·859–0·883; figure 2).

## Discussion

We have validated a clinical case-finding algorithm (FAMCAT) to improve the identification of patients at highest likelihood of having familial hypercholesterolaemia in primary care. In this large retrospective cohort study, we found that FAMCAT had high predictive accuracy to identify such cases by using routinely available variables in patients' electronic health records. FAMCAT was also more accurate than currently available and recommended approaches to identify cases of possible familial hypercholesterolaemia using diagnostic criteria or very high total cholesterol (which results in a large absolute number of patients with familial hypercholesterolaemia who are not identified). The algorithm also performed well across ethnic groups in the general primary care population, albeit with some variation in predictive accuracy among ethnic minority groups. The addition of previous personal history of premature myocardial infarction, and fitting a model with total cholesterol, LDL cholesterol, triglycerides, and age as continuous variables significantly improved FAMCAT predictive accuracy even further.

The current external validation of this algorithm has been achieved in 747 000 randomly selected patients (from the QResearch database) registered with primary care practices, independent of the CPRD database from which the FAMCAT algorithm was developed. To our knowledge, this is the only algorithm for clinical case finding of familial hypercholesterolaemia in primary care to be robustly developed and stringently validated using two separate and similarly large-sized cohort studies. Including previous development (about 2·2 million patients) and internal validation (about 742 000 patients) cohorts, FAMCAT has now been applied to almost 3·7 million patients—ie, 7% of the UK adult population.

The strong performance and consistent predictive accuracy of the FAMCAT algorithm, with high discrimination values of 0·83 (in this study) and 0·86^15^ in two separate databases, is not surprising because its development, internal validation, and current external validation have been in very large primary care patient populations. In line with other robustly developed disease prediction algorithms, we have demonstrated consistent predictive accuracy in two independent databases. This finding supports the potential benefits that FAMCAT might offer in using electronic health records to case find patients with the highest likelihood of having familial hypercholesterolaemia.

Other strengths of this study include its longitudinal design, with a long duration of follow-up, representativeness, and lack of selection, recall, and respondent bias. The QResearch database used here is, to the best of our knowledge, the world's largest primary medical care database, from which several well established clinical algorithms have been developed and validated.^21^, ^22^, ^23^

Nevertheless, we acknowledge limitations shared in common with these other risk algorithms and large database analyses.^23^, ^24^, ^25^ These limitations include lack of formal adjudication of diagnoses, information bias, and potential bias due to missing data. The specific coding of familial hypercholesterolaemia used will include individuals with a diagnosis of familial hypercholesterolaemia by a lipid specialist using clinical diagnostic criteria or genetic testing because there are currently no distinct codes for either in clinical systems. Furthermore, some patients with phenotypic familial hypercholesterolaemia might not have a monogenic mutation identified, but conditions such as polygenic familial hypercholesterolaemia or familial combined hyperlipidaemia.^26^ Concordance between phenotypic criteria (DLCN, Simon Broome, or MEDPED) and familial hypercholesterolaemia-causing mutations is moderate.^27^ FAMCAT is designed to identify cases with a clinical phenotype indicating the highest likelihood of existing disease and therefore to reduce atherosclerotic risk of such cases irrespective of a confirmed mutation. For such case finding in the general primary care population, and for validation in this context, the outcome of interest is thus the clinical phenotype for familial hypercholesterolaemia. Ascertainment bias, although not unique to this condition or database analyses, should also be noted because some patients with familial hypercholesterolaemia might be misclassified, have not yet been identified, or might not have had cholesterol assessed.^15^

We note that family history, which is a prerequisite for all familial hypercholesterolaemia case-finding approaches, is often under-reported in electronic health records and more detailed free-text notes could not be accessed because of privacy laws. Although the accuracy of FAMCAT in detecting familial hypercholesterolaemia in various ethnicities in the cohort was good, the study population mainly comprised white Europeans, and therefore its applicability to other ethnic groups needs further exploration. Finally, there was a lower prevalence of familial hypercholesterolaemia (0·2%) seen in this cohort compared with more recent evidence suggesting general population prevalence of 0·4%.^28^ This difference is probably due to general practioners offering cholesterol testing to older patients with comorbidities, and vascular and secondary risk factors as part of the NHS vascular check programme.^29^

Three other electronic health record tools exist for familial hypercholesterolaemia case finding.^30^, ^31^, ^32^ TARB-ex^32^ developed in Australia and SEARCH in the USA^31^ are based on DLCN criteria. Both apply specialist lipid clinic criteria and scoring to primary care by, for example, using natural language processing to extract family history of coronary heart disease from records.^31^ Another algorithm developed from the Dutch familial hypercholesterolaemia screening cohort showed similar discrimination to FAMCAT and was validated using a separate lipid clinic cohort.^30^ However, this study involved more selected patient populations, already referred to specialists with suspected familial hypercholesterolaemia. Other research applying specialist Simon Broome diagnostic criteria for case finding in primary care found that as many as 2·3% of all patients (more than 10-fold expected frequency of familial hypercholesterolaemia) would require referral for further assessment.^13^ By contrast, FAMCAT has been designed specifically for clinical case finding in primary care, to most effectively identify people at the highest probability of familial hypercholesterolaemia, who should then be appropriately further referred for specialist assessment and diagnosis.

With about 80% of people affected by familial hypercholesterolaemia currently undiagnosed, improving detection of this life-limiting condition should be a public health priority.^3^, ^5^ The current study underlines the poor predictive accuracy of using very elevated cholesterol concentrations alone for case finding in the general population. Other recommended approaches, such as additional use of family history of premature coronary heart disease based on Simon Broome criteria, identify too many people as having possible familial hypercholesterolaemia who will not have the condition.^13^ Internationally, use of specialist DLCN or MEDPED criteria are recommended.^11^, ^12^ However, the current study and our previous study^15^ show FAMCAT has better predictive accuracy for clinical case finding than any of these approaches, and in very large primary care populations.

In the UK, FAMCAT has become an automated tool for case finding of possible familial hypercholesterolaemia in primary care records and is being updated to reflect improvements in accuracy from using continuous lipid measurements and inclusion of personal history of premature myocardial infarction. Full release of the regression coefficients and variable code definitions will allow researchers to independently implement the algorithm within their own respective populations.

Using routine search of electronic health records, FAMCAT can be applied in real time to all adult patients registered to a general practice or clinic who have had their cholesterol measured previously. Adult patients who have a high FAMCAT probability of having familial hypercholesterolaemia can be referred for specialist assessment (see clinical examples in appendix p 4). Subsequent lipid specialist assessment of cases identified by this approach is required for diagnostic confirmation, which might also include genetic testing and screening of relatives. Once the index case is identified with familial hypercholesterolaemia, lipid therapies can be appropriately optimised (high-potency statins), and cholesterol testing and cascade screening of unidentified relatives can occur, an approach that has been shown to be cost-effective.^33^

Systematically searching electronic health records with information technology tools that identify patients at high risk could complement current opportunistic practice.^34^ Other promising methods of screening for familial hypercholesterolaemia in primary care include screening children with elevated cholesterol for familial hypercholesterolaemia and cascade testing parents.^35^ These approaches, along with FAMCAT, could potentially greatly enhance familial hypercholesterolaemia identification in the community. Our further research is prospectively evaluating whether FAMCAT improves familial hypercholesterolaemia detection rates confirmed by genetic testing and determining the cost-effectiveness of FAMCAT.

In conclusion, when applied to a large independent cohort of patients, the FAMCAT algorithm performs well and with greater accuracy than currently recommended approaches to identify familial hypercholesterolaemia in primary care. FAMCAT has great potential to improve case finding for familial hypercholesterolaemia and enhance detection of undiagnosed familial hypercholesterolaemia in the general population.
