# Correction to *Lancet Public Health* 2019; 4: e256–64

**DOI:** 10.1016/S2468-2667(19)30113-6

**Published:** 2019-07-03

*Weng S, Kai J, Akyea R, Qureshi N. Detection of familial hypercholesterolaemia: external validation of the FAMCAT clinical case-finding algorithm to identify patients in primary care.* Lancet Public Health *2019; **4:** e256–64—*In table 4 of this Article, the penultimate sentence of the footnote should read “§Simvastatin 80 mg per day, atorvastatin 20 mg or more per day, or rosuvastatin 10 mg or more per day.” The footnote for Supplemental Table 3 in the webappendix of this Article has also been corrected. These corrections have been made as of July 3, 2019.

