# Supplementary material for: Detection of familial hypercholesterolaemia: external validation of the FAMCAT clinical case-finding algorithm to identify patients in primary care
Source: Lancet Public Health. 2019 May 1;4(5):e256–64. doi: 10.1016/S2468-2667(19)30061-1 (PMC6506568; doi:10.1016/S2468-2667(19)30061-1)
Supplement: Supplementary appendix [file mmc1.pdf]

THE LANCET
Public Health

Supplementary appendix

This appendix formed part of the original submission and has been peer reviewed. We post it as supplied by the authors.

Supplement to: Weng S, Kai J, Akyea R, Qureshi N. Detection of familial hypercholesterolaemia: external validation of the FAMCAT clinical case-finding algorithm to identify patients in primary care. *Lancet Public Health* 2019; **4:** e256–64.

Supplemental Table 1. FAMCAT algorithm coefficients and constants derived from the Clinical Practice Research Datalink (CPRD) for determining probability of familial hypercholesterolaemia

Diagnostic Variables	Coefficients	
	**Men**	**Women**
**Total cholesterol or LDL-cholesterol recorded (mmol/L)**		
Ideal	0.0000	0.0000
High	0.9159	0.9557
Very High	2.0515	2.0951
Extremely High	3.6367	3.7633
**Age during cholesterol measurement (years)**		
16-24	0.0000	0.0000
25-34	-0.5653	-0.3536
35-44	-1.0734	-0.8451
45-54	-1.5090	-1.2109
55-64	-2.0856	-1.5836
65-74	-2.6143	-2.0009
75-84	-2.9906	-2.7792
85 or above	-2.9887	-3.1668
**Triglycerides during cholesterol measurement (mmol/L)**		
Ideal	0.0000	0.0000
Borderline High	-0.0593	-0.0411
High	-0.2111	-0.1612
Very High	-0.3239	-0.5468
**Lipid lowering drug usage during cholesterol measurement**		
No lipid lowering drugs prescribed	0.0000	0.0000
Other drugs	1.5683	1.4576
Low potency statins	0.9104	1.0184
Medium potency statin	1.4967	1.2584
High potency statins	2.3647	1.8423
**Family history of familial hypercholesterolaemia**		
No	0.0000	0.0000
Yes	2.3972	2.1053
**Family history of coronary heart disease**		
No	0.0000	0.0000
Yes	0.6377	0.5617
**Family history of raised cholesterol**		
No	0.0000	0.0000
Yes	1.1706	1.1719
**Diagnosis of diabetes**		
No	0.0000	0.0000
Yes	-1.1004	-0.8887
**Diagnosis of kidney disease**		
No	0.0000	0.0000
Yes	-0.4293	-0.3215
**Constant Term**	-6.5788	-6.7398

Probability of Familial Hypercholesterolaemia = $\frac{e^{xb}}{1+e^{xb}}$

where *xb* is the linear predictor of the combined coefficients and constant

Supplemental Table 2. Model discrimination in the external validation cohort for identifying familial hypercholesterolaemia in primary care, stratified by gender (n = 747,000)

Models	Men (n = 362,769)			Women (n = 384,231)		
	AUROC (c-statistic)	Standard Error[$]	95% Confidence Interval	AUROC (c-statistic)	Standard Error[$]	95% Confidence Interval
FAMCAT	0.815	0.008	0.799 – 0.831	0.848	0.007	0.834 – 0.862
Simon Broome Criteria [a]	0.673	0.011	0.651 – 0.695	0.707	0.008	0.691 – 0.723
Dutch Lipid Clinic Criteria [b]	0.703	0.011	0.681 – 0.725	0.737	0.009	0.719 – 0.755
MEDPED Criteria [c]	0.615	0.011	0.593 – 0.637	0.635	0.008	0.619 – 0.650
Cholesterol above 99th centile [d]	0.559	0.008	0.543 – 0.575	0.595	0.007	0.581 – 0.609

[$] Jack-knife procedure to estimate standard errors [20]
[a] Total cholesterol > 7.5 mmol/L or LDL-cholesterol > 4.9 mmol/L + family history of premature myocardial infarction [10]
[b] Score based on LDL-cholesterol, family history, clinical history, and physical examination [11]
[c] Age-stratified total cholesterol thresholds for the general population [12]
[d] Total cholesterol > 9.0 mmol/L or LDL-cholesterol > 6.6 mmol/L if age > 30 years; Total cholesterol > 7.5 mmol/L or LDL-cholesterol > 4.9 mmol/L if age ≤ 30 years [10]

Supplemental Table 3. FAMCAT optimised algorithm coefficients and constants derived from the QRESEARCH for determining probability of familial hypercholesterolaemia

**Diagnostic Variables**	**Coefficients**	
	**Men**	**Women**
Highest cholesterol recorded (mmol/L)		
If LDL Cholesterol measured	0.9458	1.1927
If Total Cholesterol measured	0.5308	0.6702
Age during cholesterol measurements (years)	-0.0297	-0.0079
Log triglycerides during cholesterol measurement (mmol/L)	-1.8518	-2.5594
Lipid lowering drugs prescribed during cholesterol measurement *		
Prescribed fibrate, bile acid sequestrant, or nicotinic acid [a]	1.9494	1.3822
Prescribed low potency statin [b]	0.0323	1.2637
Prescribed medium potency statin [c]	0.4466	0.5440
Prescribed high potency statin [d]	0.5649	0.9350
Previously history of premature myocardial infarction*	0.8319	0.4337
Family history of familial hypercholesterolaemia*	1.9076	0.9109
Family history of myocardial infarction* [+]	1.2621	1.4754
Family history of raised cholesterol*	1.0297	0.7521
Diagnosis of diabetes*	-1.3997	-0.7738
Diagnosis of chronic kidney disease*	-0.2528	-1.7661
Constant	-8.1134	-10.0826

***** Reference group = none ($\beta = 1$); [a] Fluvastatin/Pravastatin 40 mg/day, Simvastatin 10 mg/day; [b] Fluvastatin/Pravastatin 80 mg/day, Simvastatin 20-40 mg/day, Atorvastatin 10 mg/day, Rosuvastatin 5 mg; [c] Simvastatin 80 mg, Atorvastatin 20 mg/day, Rosuvastatin 10 mg/day; [+] premature defined as < 55 years in men and < 60 years in women

Probability of Familial Hypercholesterolaemia = $\frac{e^{xb}}{1+e^{xb}}$

where *xb* is the linear predictor of the combined coefficients and constant

Supplemental Table 4. Clinical examples using FAMCAT for case-finding in primary care compared to standard guidance

FAMCAT ranking of patients' likelihood of FH	Age	Sex	LDL Cholesterol (mmol/L)	Triglycerides (mmol/L)	Family History	Lipid Lowering Drug	Secondary Causes	FAMCAT Relative Risk*	Meets NICE-recommended Simon Broome criteria for referral[11]	Primary Care *Decision after applying FAMCAT*
1st	43	M	5.1	1.3	MI < 60 y, Raised Cholesterol	Simvastatin 40 mg/day	None	164.74	Yes	*Refer to lipid specialist*
5th	33	M	4.7	0.8	MI < 60 y, Raised Cholesterol	None	None	18.06	No	*Refer to lipid specialist despite not meeting Simon Broome criteria*
21st	51	F	4.5	2.0	MI < 60 y	Simvastatin 10 mg/day	None	6.58	No	*Refer to lipid specialist despite not meeting Simon Broome criteria*
441st	66	M	5.5	4.8	MI < 60 y	None	Type 2 diabetes	0.98	Yes	*Although meets Simon Broome criteria, not referred - low FAMCAT rank, note diabetes & raised triglycerides)*

* Relative risk is calculated by dividing the probability of FH generated by FAMCAT regression equations by 0.002 (estimated 1/500 prevalence of FH in the general population)

FAMCAT algorithm (regression equations) is applied to the general practice electronic health records of all individuals who have a recorded lipids measurement. Each patient in a practice is then individually ranked by this tool from highest to lowest likelihood of FH. The FAMCAT algorithm can be found at https://prism-uon.shinyapps.io/FAMCAT/ for general population usage. For UK general practices, there is also an integrated case-finding tool. [15] This is currently freely available to practices using EMIS or SystmOne/TPP as a quality improvement tool (https://www.nottingham.ac.uk/primis/tools-audits/tools-audits/familial-hypercholesterolaemia.aspx).

Online Supplement: FAMCAT web based calculator (linked to calculator provided below)

Familial Hypercholesterolaemia Case Ascertainment Tool (FAMCAT)

Primary Care Stratified Medicine

University of Nottingham UK | CHINA | MALAYSIA

ENTER/SELECT DETAILS BELOW:

Gender

Male

Age at the time cholesterol measured (years)

16

Total Cholesterol (mmol/L)

0

LDL Cholesterol (mmol/L)

0

Triglycerides (mmol/L)

0

On lipid lowering drug therapy when cholesterol measured

None

Family history of familial hypercholesterolaemia

No

Family history of myocardial infarction

No

Family history of raised cholesterol

No

Previously diagnosed with diabetes

No

Previously diagnosed with chronic kidney disease

No

Welcome to the FAMCAT online risk calculator
You can use this calculator to work out the likelihood of an individual having an inherited condition called ***Familial Hypercholesterolaemia***. This a common inherited cause of raised cholesterol, affecting at least 1 in 500 individuals in the general population. However, up 80% of people with FH are still not identified in many countries, leading to many avoidable heart attacks and early deaths. The risk of heart disease can be dramatically reduced by starting medicines to lower cholesterol levels.

The FAMCAT calculator determines the likelihood of having familial hypercholesterolaemia. This is based on calculating a probability value using data entered in this application. The algorithm then estimates a ***relative population risk***.

A relative population risk < 1 means the individual is unlikely to have familial hypercholesterolaemia. We suggest keeping family history updated regularly

A relative population risk from 1 - 5 means the individual may have familial hypercholesterolaemia. We suggest confirming the family history is correct and consider referral to lipid specialists following clinical guidelines

A relative population risk from > 5 means the individual is likely to have familial hypercholesterolaemia. We suggest referral to lipid specialists and considering genetic testing following clinical guidelines

FAMCAT Calculator	Probability (%)	Relative Population Risk
Likelihood of having familial hypercholesterolaemia	0.00	0.27

Disclaimer: The FAMCAT algorithm has been developed by the Primary Care Stratified Medicine (PRISM) team based in the Division of Primary Care at the University of Nottingham. The algorithm has been developed and validated using patient data which has been routinely collected by doctors and nurses in UK family practices contributing to the Clinical Practice Research Datalink. Further, the algorithm has now been externally validated using another routinely collected database of entirely separate UK family practices contributing to QRESEARCH. All medical decisions need to be taken in consultation with a licenced health care professional. The authors accept no responsibility for clinical use or misuse of this tool.

Publication: Full details of the algorithm development and validation can be found in peer-reviewed journal *Atherosclerosis*

Copyright: Primary Care Stratified Medicine, University of Nottingham 2018. All rights reserved

Uselink Links: Guidelines for identification and management of familial hypercholesterolaemia

UK: National Institute for Health and Care Excellence

Europe: ESC/EAS Task Force

US: ACC/AHA Task Force

Contact Details: Dr Stephen Weng, Assistant Professor of Integrative Epidemiology and Data Science, University of Nottingham, stephen.weng@nottingham.ac.uk

https://prism-uon.shinyapps.io/FAMCAT/

Supplementary – Code List of All Variables Included in the Model

1) **Lipid Measurements**

Total Cholesterol

44OE	Plasma total cholesterol level
44P	Serum cholesterol
44P1	Serum cholesterol normal
44P2	Serum cholesterol borderline
44P3	Serum cholesterol raised
44P4	Serum cholesterol very higha
44PH	Total cholesterol measurement
44PJ	Serum total cholesterol level
44PK	Serum fasting total cholesterol
44PZ	Serum cholesterol NOS
4I3O	Fluid sample cholesterol level
EGTON456	Fasting serum cholesterol

LDL Cholesterol

44d4	Plasma random LDL cholesterol level
44d5	Plasma fasting LDL cholesterol level
44dB	Plasma LDL cholesterol level
44P6	Serum LDL cholesterol level
44P7	Serum VLDL cholesterol level
44PD	Serum fasting LDL cholesterol level
44PE	Serum random LDL cholesterol level
44PI	Calculated LDL cholesterol level
44PL	Non HDL cholesterol level
44PL0	Serum non high density lipoprotein cholesterol level
44PL1	Estimated serum non-HDL cholesterol level
EGTONLD1	LDL cholesterol level

Triglycerides

44e	Plasma triglyceride level
44e0	Plasma random triglyceride level
44e1	Plasma fasting triglyceride level
44Q	Serum triglycerides
44Q1	Serum triglycerides normal
44Q2	Serum triglycerides borderline
44Q3	Serum triglycerides raised
44Q4	Serum fasting triglyceride level
44Q5	Serum random triglyceride level
44QZ	Serum triglycerides NOS
4I3W	Fluid sample triglyceride level
4QA1	Triglyceride level

EGTON457	Fasting triglycerides

2) Definite Familial Hypercholesterolaemia

C320-1	Familial hypercholesterolaemia
C3200	Familial hypercholesterolaemia

3) Family History of Familial Hypercholesterolaemia

1269	Family history of familial hypercholesterolaemia

4) Family History of Raised Cholesterol/Hypercholesterolaemia

122H	No FH of hypercholesterolaemia
1262	FH: Raised blood lipids
126B	FH: Hypercholesterolaemia in first degree relative

5) Family History of myocardial infarction

12C2-1	FH: Myocardial infarction < 60
12C2-2	FH: MI- Myocardial infarct <60
12C3-1	FH: Myocardial infarction > 60
12C3-2	FH: MI- myocardial infarct >60
12C5	FH: Myocardial infarction
12C5-2	FH: Ischaemic heart disease
12CA	FH myocardial infarction male first degree age known
12CB	FH myocardial infarction male first degree age unknown
12CC	FH myocardial infarction female first degree age known
12CD	FH myocardial infarction female first degree age unknown
12CI	FH: premature coronary heart disease
12CN	FH: Myocardial infarct in 1st degree female relative <65 yrs
12CP	FH: Myocardial infarct in 1st degree male relative <55 years
ZV173	[V]Family history of ischaemic heart disease
ZV173-1	[V]Family history of ischaemic heart disease (IHD)
ZV173-2	[V]Family history of myocardial infarction

6) History of cardiovascular disease

Coronary Heart Disease

G3	Ischaemic heart disease
G3-1	Arteriosclerotic heart disease
G3-2	Atherosclerotic heart disease
G3-3	IHD - Ischaemic heart disease
G30	Acute myocardial infarction
G30-1	Attack - heart
G30-2	Coronary thrombosis

G30-3	Cardiac rupture following myocardial infarction (MI)
G30-4	Heart attack
G30-5	MI - acute myocardial infarction
G30-6	Thrombosis - coronary
G30-7	Silent myocardial infarction
G300	Acute anterolateral infarction
G301	Other specified anterior myocardial infarction
G3010	Acute anteroapical infarction
G3011	Acute anteroseptal infarction
G301z	Anterior myocardial infarction NOS
G302	Acute inferolateral infarction
G303	Acute inferoposterior infarction
G304	Posterior myocardial infarction NOS
G305	Lateral myocardial infarction NOS
G306	True posterior myocardial infarction
G307	Acute subendocardial infarction
G3070	Acute non-Q wave infarction
G3071	Acute non-ST segment elevation myocardial infarction
G308	Inferior myocardial infarction NOS
G309	Acute Q-wave infarct
G30B	Acute posterolateral myocardial infarction
G30X	Acute transmural myocardial infarction of unspecif site
G30X0	Acute ST segment elevation myocardial infarction
G30y	Other acute myocardial infarction
G30y0	Acute atrial infarction
G30y1	Acute papillary muscle infarction
G30y2	Acute septal infarction
G30yz	Other acute myocardial infarction NOS
G30z	Acute myocardial infarction NOS
G31	Other acute and subacute ischaemic heart disease
G310	Postmyocardial infarction syndrome
G310-1	Dressler's syndrome
G311	Preinfarction syndrome
G311-1	Crescendo angina
G311-2	Impending infarction
G311-3	Unstable angina
G311-4	Angina at rest
G3110	Myocardial infarction aborted
G3110-1	MI - myocardial infarction aborted
G3111	Unstable angina
G3112	Angina at rest
G3113	Refractory angina
G3114	Worsening angina
G3115	Acute coronary syndrome

G311z	Preinfarction syndrome NOS
G312	Coronary thrombosis not resulting in myocardial infarction
G31y	Other acute and subacute ischaemic heart disease
G31y0	Acute coronary insufficiency
G31y2	Subendocardial ischaemia
G31y3	Transient myocardial ischaemia
G31yz	Other acute and subacute ischaemic heart disease NOS
G32	Old myocardial infarction
G32-1	Healed myocardial infarction
G32-2	Personal history of myocardial infarction
G33	Angina pectoris
G330	Angina decubitus
G3300	Nocturnal angina
G330z	Angina decubitus NOS
G33z	Angina pectoris NOS
G33z0	Status anginosus
G33z1	Stenocardia
G33z2	Syncope anginosa
G33z3	Angina on effort
G33z4	Ischaemic chest pain
G33z5	Post infarct angina
G33z6	New onset angina
G33z7	Stable angina
G33zz	Angina pectoris NOS
G34	Other chronic ischaemic heart disease
G340	Coronary atherosclerosis
G340-1	Triple vessel disease of the heart
G340-2	Coronary artery disease
G3400	Single coronary vessel disease
G3401	Double coronary vessel disease
G342	Atherosclerotic cardiovascular disease
G344	Silent myocardial ischaemia
G34y	Other specified chronic ischaemic heart disease
G34y0	Chronic coronary insufficiency
G34y1	Chronic myocardial ischaemia
G34yz	Other specified chronic ischaemic heart disease NOS
G34z	Other chronic ischaemic heart disease NOS
G34z0	Asymptomatic coronary heart disease
G35	Subsequent myocardial infarction
G350	Subsequent myocardial infarction of anterior wall
G351	Subsequent myocardial infarction of inferior wall
G353	Subsequent myocardial infarction of other sites
G35X	Subsequent myocardial infarction of unspecified site
G36	Certain current complication follow acute myocardial infarct

G360	Haemopericardium/current comp folow acut myocard infarct
G361	Atrial septal defect/curr comp folow acut myocardal infarct
G362	Ventric septal defect/curr comp fol acut myocardal infarctn
G363	Ruptur cardiac wall w'out haemopericard/cur comp fol ac MI
G364	Ruptur chordae tendinae/curr comp fol acute myocard infarct
G365	Rupture papillary muscle/curr comp fol acute myocard infarct
G366	Thrombosis atrium,auric append&vent/curr comp foll acute MI
G38	Postoperative myocardial infarction
G380	Postoperative transmural myocardial infarction anterior wall
G381	Postoperative transmural myocardial infarction inferior wall
G384	Postoperative subendocardial myocardial infarction
G38z	Postoperative myocardial infarction, unspecified

Cerebrovascular Accident

G61-2	Stroke due to intracerebral haemorrhage
G64-3	Stroke due to cerebral arterial occlusion
G65-2	Transient ischaemic attack
G66	Stroke and cerebrovascular accident unspecified
G66-2	Stroke unspecified
G663	Brain stem stroke syndrome
G664	Cerebellar stroke syndrome
G68X	Sequelae of stroke,not specfd as h'morrhage or infarction
Gyu30	[X]Other forms of angina pectoris
Gyu34	[X]Acute transmural myocardial infarction of unspecif site

7) History of Peripheral Vascular Disease

G73	Other peripheral vascular disease
G73y	Other specified peripheral vascular disease
G73yz	Other specified peripheral vascular disease NOS
G73z	Peripheral vascular disease NOS
G73zz	Peripheral vascular disease NOS
Gyu74	[X]Other specified peripheral vascular diseases

8) History of Diabetes

1JL	Suspected diabetes mellitus
66AJ-1	Unstable diabetes
66AJ1	Brittle diabetes
C10	Diabetes mellitus
C100	Diabetes mellitus with no mention of complication
C1000	Diabetes mellitus, juvenile type, no mention of complication
C1000-1	Insulin dependent diabetes mellitus
C1001	Diabetes mellitus, adult onset, no mention of complication
C1001-1	Maturity onset diabetes

C1001-2	Non-insulin dependent diabetes mellitus
C100z	Diabetes mellitus NOS with no mention of complication
C101	Diabetes mellitus with ketoacidosis
C1010	Diabetes mellitus, juvenile type, with ketoacidosis
C1011	Diabetes mellitus, adult onset, with ketoacidosis
C101y	Other specified diabetes mellitus with ketoacidosis
C101z	Diabetes mellitus NOS with ketoacidosis
C102	Diabetes mellitus with hyperosmolar coma
C1020	Diabetes mellitus, juvenile type, with hyperosmolar coma
C1021	Diabetes mellitus, adult onset, with hyperosmolar coma
C102z	Diabetes mellitus NOS with hyperosmolar coma
C103	Diabetes mellitus with ketoacidotic coma
C1030	Diabetes mellitus, juvenile type, with ketoacidotic coma
C1031	Diabetes mellitus, adult onset, with ketoacidotic coma
C103y	Other specified diabetes mellitus with coma
C103z	Diabetes mellitus NOS with ketoacidotic coma
C104	Diabetes mellitus with renal manifestation
C1040	Diabetes mellitus, juvenile type, with renal manifestation
C1041	Diabetes mellitus, adult onset, with renal manifestation
C104y	Other specified diabetes mellitus with renal complications
C104z	Diabetes mellitus with nephropathy NOS
C105	Diabetes mellitus with ophthalmic manifestation
C1050	Diabetes mellitus, juvenile type, + ophthalmic manifestation
C1051	Diabetes mellitus, adult onset, + ophthalmic manifestation
C105y	Other specified diabetes mellitus with ophthalmic complicatn
C105z	Diabetes mellitus NOS with ophthalmic manifestation
C106	Diabetes mellitus with neurological manifestation
C106-2	Diabetes mellitus with neuropathy
C106-3	Diabetes mellitus with polyneuropathy
C1060	Diabetes mellitus, juvenile, + neurological manifestation
C1061	Diabetes mellitus, adult onset, + neurological manifestation
C106y	Other specified diabetes mellitus with neurological comps
C106z	Diabetes mellitus NOS with neurological manifestation
C107	Diabetes mellitus with peripheral circulatory disorder
C107-1	Diabetes mellitus with gangrene
C107-2	Diabetes with gangrene
C1070	Diabetes mellitus, juvenile +peripheral circulatory disorder
C1071	Diabetes mellitus, adult, + peripheral circulatory disorder
C1072	Diabetes mellitus, adult with gangrene
C107z	Diabetes mellitus NOS with peripheral circulatory disorder
C108	Insulin dependent diabetes mellitus
C108-1	IDDM-Insulin dependent diabetes mellitus
C108-2	Type 1 diabetes mellitus
C108-3	Type I diabetes mellitus

C1080	Insulin-dependent diabetes mellitus with renal complications
C1080-1	Type I diabetes mellitus with renal complications
C1080-2	Type 1 diabetes mellitus with renal complications
C1081	Insulin-dependent diabetes mellitus with ophthalmic comps
C1081-2	Type 1 diabetes mellitus with ophthalmic complications
C1082	Insulin-dependent diabetes mellitus with neurological comps
C1082-1	Type I diabetes mellitus with neurological complications
C1082-2	Type 1 diabetes mellitus with neurological complications
C1083	Insulin dependent diabetes mellitus with multiple complicatn
C1084	Unstable insulin dependent diabetes mellitus
C1084-1	Unstable type I diabetes mellitus
C1084-2	Unstable type 1 diabetes mellitus
C1085	Insulin dependent diabetes mellitus with ulcer
C1085-1	Type I diabetes mellitus with ulcer
C1085-2	Type 1 diabetes mellitus with ulcer
C1086	Insulin dependent diabetes mellitus with gangrene
C1087	Insulin dependent diabetes mellitus with retinopathy
C1087-1	Type I diabetes mellitus with retinopathy
C1087-2	Type 1 diabetes mellitus with retinopathy
C1088	Insulin dependent diabetes mellitus - poor control
C1088-1	Type I diabetes mellitus - poor control
C1088-2	Type 1 diabetes mellitus - poor control
C1089	Insulin dependent diabetes maturity onset
C1089-1	Type I diabetes mellitus maturity onset
C1089-2	Type 1 diabetes mellitus maturity onset
C108A	Insulin-dependent diabetes without complication
C108A-1	Type I diabetes mellitus without complication
C108B	Insulin dependent diabetes mellitus with mononeuropathy
C108B-1	Type I diabetes mellitus with mononeuropathy
C108C	Insulin dependent diabetes mellitus with polyneuropathy
C108D	Insulin dependent diabetes mellitus with nephropathy
C108D-1	Type I diabetes mellitus with nephropathy
C108E	Insulin dependent diabetes mellitus with hypoglycaemic coma
C108E-1	Type I diabetes mellitus with hypoglycaemic coma
C108E-2	Type 1 diabetes mellitus with hypoglycaemic coma
C108F	Insulin dependent diabetes mellitus with diabetic cataract
C108F-1	Type I diabetes mellitus with diabetic cataract
C108H	Insulin dependent diabetes mellitus with arthropathy
C108H-1	Type I diabetes mellitus with arthropathy
C108J-1	Type I diabetes mellitus with neuropathic arthropathy
C108J-2	Type 1 diabetes mellitus with neuropathic arthropathy
C108y	Other specified diabetes mellitus with multiple comps
C108z	Unspecified diabetes mellitus with multiple complications
C109	Non-insulin dependent diabetes mellitus

C109-1	NIDDM - Non-insulin dependent diabetes mellitus
C109-2	Type 2 diabetes mellitus
C109-3	Type II diabetes mellitus
C1090	Non-insulin-dependent diabetes mellitus with renal comps
C1090-1	Type II diabetes mellitus with renal complications
C1090-2	Type 2 diabetes mellitus with renal complications
C1091	Non-insulin-dependent diabetes mellitus with ophthalm comps
C1091-1	Type II diabetes mellitus with ophthalmic complications
C1091-2	Type 2 diabetes mellitus with ophthalmic complications
C1092	Non-insulin-dependent diabetes mellitus with neuro comps
C1092-1	Type II diabetes mellitus with neurological complications
C1092-2	Type 2 diabetes mellitus with neurological complications
C1093	Non-insulin-dependent diabetes mellitus with multiple comps
C1094	Non-insulin dependent diabetes mellitus with ulcer
C1094-1	Type II diabetes mellitus with ulcer
C1094-2	Type 2 diabetes mellitus with ulcer
C1095	Non-insulin dependent diabetes mellitus with gangrene
C1095-1	Type II diabetes mellitus with gangrene
C1095-2	Type 2 diabetes mellitus with gangrene
C1096	Non-insulin-dependent diabetes mellitus with retinopathy
C1096-1	Type II diabetes mellitus with retinopathy
C1096-2	Type 2 diabetes mellitus with retinopathy
C1097	Non-insulin dependent diabetes mellitus - poor control
C1097-1	Type II diabetes mellitus - poor control
C1097-2	Type 2 diabetes mellitus - poor control
C1099	Non-insulin-dependent diabetes mellitus without complication
C109A	Non-insulin dependent diabetes mellitus with mononeuropathy
C109A-1	Type II diabetes mellitus with mononeuropathy
C109B	Non-insulin dependent diabetes mellitus with polyneuropathy
C109B-1	Type II diabetes mellitus with polyneuropathy
C109C	Non-insulin dependent diabetes mellitus with nephropathy
C109C-1	Type II diabetes mellitus with nephropathy
C109C-2	Type 2 diabetes mellitus with nephropathy
C109D	Non-insulin dependent diabetes mellitus with hypoglyca coma
C109D-1	Type II diabetes mellitus with hypoglycaemic coma
C109D-2	Type 2 diabetes mellitus with hypoglycaemic coma
C109E	Non-insulin depend diabetes mellitus with diabetic cataract
C109E-1	Type II diabetes mellitus with diabetic cataract
C109E-2	Type 2 diabetes mellitus with diabetic cataract
C109F-1	Type II diabetes mellitus with peripheral angiopathy
C109F-2	Type 2 diabetes mellitus with peripheral angiopathy
C109G	Non-insulin dependent diabetes mellitus with arthropathy
C109G-1	Type II diabetes mellitus with arthropathy
C109G-2	Type 2 diabetes mellitus with arthropathy

C109H-1	Type II diabetes mellitus with neuropathic arthropathy
C109H-2	Type 2 diabetes mellitus with neuropathic arthropathy
C109J	Insulin treated Type 2 diabetes mellitus
C109J-1	Insulin treated non-insulin dependent diabetes mellitus
C109J-2	Insulin treated Type II diabetes mellitus
C10A5	Malnutritn-relat diabetes melitus wth periph circul complctn
C10B	Diabetes mellitus induced by steroids
C10B0	Steroid induced diabetes mellitus without complication
C10C	Diabetes mellitus autosomal dominant
C10C-1	Maturity onset diabetes in youth
C10C-2	Maturity onset diabetes in youth type 1
C10D	Diabetes mellitus autosomal dominant type 2
C10D-1	Maturity onset diabetes in youth type 2
C10E	Type 1 diabetes mellitus
C10E-1	Type I diabetes mellitus
C10E-2	Insulin dependent diabetes mellitus
C10E0	Type 1 diabetes mellitus with renal complications
C10E0-2	Insulin-dependent diabetes mellitus with renal complications
C10E1	Type 1 diabetes mellitus with ophthalmic complications
C10E1-1	Type I diabetes mellitus with ophthalmic complications
C10E1-2	Insulin-dependent diabetes mellitus with ophthalmic comps
C10E2	Type 1 diabetes mellitus with neurological complications
C10E2-2	Insulin-dependent diabetes mellitus with neurological comps
C10E3	Type 1 diabetes mellitus with multiple complications
C10E3-1	Type I diabetes mellitus with multiple complications
C10E3-2	Insulin dependent diabetes mellitus with multiple complicat
C10E4	Unstable type 1 diabetes mellitus
C10E4-1	Unstable type I diabetes mellitus
C10E4-2	Unstable insulin dependent diabetes mellitus
C10E5	Type 1 diabetes mellitus with ulcer
C10E5-1	Type I diabetes mellitus with ulcer
C10E5-2	Insulin dependent diabetes mellitus with ulcer
C10E6	Type 1 diabetes mellitus with gangrene
C10E6-1	Type I diabetes mellitus with gangrene
C10E7	Type 1 diabetes mellitus with retinopathy
C10E7-1	Type I diabetes mellitus with retinopathy
C10E7-2	Insulin dependent diabetes mellitus with retinopathy
C10E8	Type 1 diabetes mellitus - poor control
C10E8-1	Type I diabetes mellitus - poor control
C10E8-2	Insulin dependent diabetes mellitus - poor control
C10E9	Type 1 diabetes mellitus maturity onset
C10E9-1	Type I diabetes mellitus maturity onset
C10EA	Type 1 diabetes mellitus without complication
C10EA-1	Type I diabetes mellitus without complication

C10EA-2	Insulin-dependent diabetes without complication
C10EB	Type 1 diabetes mellitus with mononeuropathy
C10EC	Type 1 diabetes mellitus with polyneuropathy
C10EC-1	Type I diabetes mellitus with polyneuropathy
C10EC-2	Insulin dependent diabetes mellitus with polyneuropathy
C10ED	Type 1 diabetes mellitus with nephropathy
C10ED-2	Insulin dependent diabetes mellitus with nephropathy
C10EE	Type 1 diabetes mellitus with hypoglycaemic coma
C10EE-2	Insulin dependent diabetes mellitus with hypoglycaemic coma
C10EF	Type 1 diabetes mellitus with diabetic cataract
C10EF-2	Insulin dependent diabetes mellitus with diabetic cataract
C10EG	Type 1 diabetes mellitus with peripheral angiopathy
C10EH	Type 1 diabetes mellitus with arthropathy
C10EJ	Type 1 diabetes mellitus with neuropathic arthropathy
C10EK	Type 1 diabetes mellitus with persistent proteinuria
C10EL	Type 1 diabetes mellitus with persistent microalbuminuria
C10EL-1	Type I diabetes mellitus with persistent microalbuminuria
C10EM	Type 1 diabetes mellitus with ketoacidosis
C10EM-1	Type I diabetes mellitus with ketoacidosis
C10EN	Type 1 diabetes mellitus with ketoacidotic coma
C10EN-1	Type I diabetes mellitus with ketoacidotic coma
C10EP	Type 1 diabetes mellitus with exudative maculopathy
C10EP-1	Type I diabetes mellitus with exudative maculopathy
C10EQ	Type 1 diabetes mellitus with gastroparesis
C10ER	Latent autoimmune diabetes mellitus in adult
C10F	Type 2 diabetes mellitus
C10F-1	Type II diabetes mellitus
C10F0	Type 2 diabetes mellitus with renal complications
C10F0-1	Type II diabetes mellitus with renal complications
C10F1	Type 2 diabetes mellitus with ophthalmic complications
C10F1-1	Type II diabetes mellitus with ophthalmic complications
C10F2	Type 2 diabetes mellitus with neurological complications
C10F2-1	Type II diabetes mellitus with neurological complications
C10F3	Type 2 diabetes mellitus with multiple complications
C10F4	Type 2 diabetes mellitus with ulcer
C10F4-1	Type II diabetes mellitus with ulcer
C10F5	Type 2 diabetes mellitus with gangrene
C10F5-1	Type II diabetes mellitus with gangrene
C10F6	Type 2 diabetes mellitus with retinopathy
C10F6-1	Type II diabetes mellitus with retinopathy
C10F7	Type 2 diabetes mellitus - poor control
C10F7-1	Type II diabetes mellitus - poor control
C10F9	Type 2 diabetes mellitus without complication
C10F9-1	Type II diabetes mellitus without complication

C10FA	Type 2 diabetes mellitus with mononeuropathy
C10FA-1	Type II diabetes mellitus with mononeuropathy
C10FB	Type 2 diabetes mellitus with polyneuropathy
C10FB-1	Type II diabetes mellitus with polyneuropathy
C10FC	Type 2 diabetes mellitus with nephropathy
C10FC-1	Type II diabetes mellitus with nephropathy
C10FD	Type 2 diabetes mellitus with hypoglycaemic coma
C10FD-1	Type II diabetes mellitus with hypoglycaemic coma
C10FE	Type 2 diabetes mellitus with diabetic cataract
C10FE-1	Type II diabetes mellitus with diabetic cataract
C10FF	Type 2 diabetes mellitus with peripheral angiopathy
C10FF-1	Type II diabetes mellitus with peripheral angiopathy
C10FG	Type 2 diabetes mellitus with arthropathy
C10FG-1	Type II diabetes mellitus with arthropathy
C10FH	Type 2 diabetes mellitus with neuropathic arthropathy
C10FJ	Insulin treated Type 2 diabetes mellitus
C10FJ-1	Insulin treated Type II diabetes mellitus
C10FL	Type 2 diabetes mellitus with persistent proteinuria
C10FL-1	Type II diabetes mellitus with persistent proteinuria
C10FM	Type 2 diabetes mellitus with persistent microalbuminuria
C10FM-1	Type II diabetes mellitus with persistent microalbuminuria
C10FN	Type 2 diabetes mellitus with ketoacidosis
C10FP	Type 2 diabetes mellitus with ketoacidotic coma
C10FQ	Type 2 diabetes mellitus with exudative maculopathy
C10FR	Type 2 diabetes mellitus with gastroparesis
C10G	Secondary pancreatic diabetes mellitus
C10G0	Secondary pancreatic diabetes mellitus without complication
C10H	Diabetes mellitus induced by non-steroid drugs
C10M	Lipoatrophic diabetes mellitus
C10N	Secondary diabetes mellitus
C10N0	Secondary diabetes mellitus without complication
C10y	Diabetes mellitus with other specified manifestation
C10y1	Diabetes mellitus, adult, + other specified manifestation
C10yy	Other specified diabetes mellitus with other spec comps
C10yz	Diabetes mellitus NOS with other specified manifestation
C10z	Diabetes mellitus with unspecified complication
C10z0	Diabetes mellitus, juvenile type, + unspecified complication
C10z1	Diabetes mellitus, adult onset, + unspecified complication
C10zy	Other specified diabetes mellitus with unspecified comps
C10zz	Diabetes mellitus NOS with unspecified complication
C11y0	Steroid induced diabetes
C314-1	Renal diabetes
C3500-1	Bronzed diabetes
Cyu2	[X]Diabetes mellitus

Cyu20	[X]Other specified diabetes mellitus
Cyu23	[X]Unspecified diabetes mellitus with renal complications
K01x1	Nephrotic syndrome in diabetes mellitus
K081	Nephrogenic diabetes insipidus
L1808-1	Gestational diabetes mellitus
L180X	Pre-existing diabetes mellitus, unspecified

9) History of Kidney Disease

1Z1	Chronic renal impairment
1Z10	Chronic kidney disease stage 1
1Z11	Chronic kidney disease stage 2
1Z12	Chronic kidney disease stage 3
1Z13	Chronic kidney disease stage 4
1Z14	Chronic kidney disease stage 5
1Z15	Chronic kidney disease stage 3A
1Z16	Chronic kidney disease stage 3B
1Z17	Chronic kidney disease stage 1 with proteinuria
1Z18	Chronic kidney disease stage 1 without proteinuria
1Z19	Chronic kidney disease stage 2 with proteinuria
1Z1A	Chronic kidney disease stage 2 without proteinuria
1Z1B	Chronic kidney disease stage 3 with proteinuria
1Z1C	Chronic kidney disease stage 3 without proteinuria
1Z1D	Chronic kidney disease stage 3A with proteinuria
1Z1E	Chronic kidney disease stage 3A without proteinuria
1Z1F	Chronic kidney disease stage 3B with proteinuria
1Z1G	Chronic kidney disease stage 3B without proteinuria
1Z1H	Chronic kidney disease stage 4 with proteinuria
1Z1J	Chronic kidney disease stage 4 without proteinuria
1Z1K	Chronic kidney disease stage 5 with proteinuria
1Z1L	Chronic kidney disease stage 5 without proteinuria
7A312	Open embolisation of renal artery
9Ot5	Predicted stage chronic kidney disease
D2150	Anaemia secondary to chronic renal failure
D4104	Renal polycythaemia
G22	Hypertensive renal disease
G222	Hypertensive renal disease with renal failure
G233	Hypertensive heart and renal disease with renal failure
G23z	Hypertensive heart and renal disease NOS
G701	Renal artery atherosclerosis
G721	Aneurysm of renal artery
G763	Hyperplasia of renal artery
K0	Nephritis, nephrosis and nephrotic syndrome
K01	Nephrotic syndrome
K010	Nephrotic syndrome with proliferative glomerulonephritis

K011	Nephrotic syndrome with membranous glomerulonephritis
K012	Nephrotic syndrome+membranoproliferative glomerulonephritis
K013	Nephrotic syndrome with minimal change glomerulonephritis
K013-2	Steroid sensitive nephrotic syndrome
K014	Nephrotic syndrome, minor glomerular abnormality
K015	Nephrotic syndrome, focal and segmental glomerular lesions
K016	Nephrotic syndrome, diffuse membranous glomerulonephritis
K017	Nephrotic syn difus mesangial prolifertiv glomerulonephritis
K018	Nephrotic syn,difus endocapilary proliftv glomerulonephritis
K01A	Nephrotic syndrome, dense deposit disease
K01B	Nephrotic syndrome, diffuse crescentic glomerulonephritis
K01w	Congenital nephrotic syndrome
K01x0	Nephrotic syndrome in amyloidosis
K01x1	Nephrotic syndrome in diabetes mellitus
K01x2	Nephrotic syndrome in malaria
K01x3	Nephrotic syndrome in polyarteritis nodosa
K01x4	Nephrotic syndrome in systemic lupus erythematosus
K01y	Nephrotic syndrome with other pathological kidney lesions
K01z	Nephrotic syndrome NOS
K034	Renal cortical necrosis unspecified
K035	Renal medullary necrosis unspecified
K04	Acute renal failure
K041	Acute renal cortical necrosis
K042	Acute renal medullary necrosis
K043	Acute drug-induced renal failure
K044	Acute renal failure due to urinary obstruction
K04y	Other acute renal failure
K05	Chronic renal failure
K05-2	End stage renal failure
K050	End stage renal failure
K06	Renal failure unspecified
K060	Renal impairment
K060-1	Impaired renal function
K071	Renal fibrosis
K07z	Renal sclerosis NOS
K08	Impaired renal function disorder
K080	Renal osteodystrophy
K08y	Other impaired renal function disorder
K08y4	Renal tubular acidosis
K08yz	Other impaired renal function disorder NOS
K08yz-1	Renal acidaemia
K08z	Impaired renal function disorder NOS
K0A07	Acute nephrotic syndrm diffuse crescentic glomerulonephritis
K0B	Renal tubulo-interstitial disorders in diseases EC

K0B40	Renal tubulo-interstitial disorder in SLE
K0D	End-stage renal disease
K0E	Acute-on-chronic renal failure
K0y	Other specified nephritis, nephrosis or nephrotic syndrome
K0z	Nephritis, nephrosis and nephrotic syndrome NOS
K138-1	Renal vascular disorders
K1380	Renal artery embolism
K1381	Renal artery haemorrhage
K1382	Renal artery thrombosis
K138z	Renal vascular disorders NOS
K138z-1	Renal infarction
Kyu1	[X]Renal tubulo-interstitial diseases
Kyu2	[X]Renal failure
Kyu20	[X]Other acute renal failure
Kyu21	[X]Other chronic renal failure
Kyu40	[X]Other disorders resulting/impaired renal tubular function
L0703	Unspecified abortion with renal failure
L093	Renal failure following abortive pregnancy
L162	Unspecified renal disease in pregnancy
L1620	Unspecified renal disease in pregnancy unspecified
L1621	Unspecified renal disease in pregnancy - delivered
L393	Acute renal failure following labour and delivery
L3930	Post-delivery acute renal failure unspecified
L3931	Post-delivery acute renal failure - delivered with p/n prob
L3932	Post-delivery acute renal failure with postnatal problem
P7690	Renal artery stenosis
PD0	Renal agenesis and dysgenesis
PD000	Bilateral renal agenesis
PD030	Bilateral renal hypoplasia
PD1	Congenital cystic kidney disease
PD1-1	Congenital cystic renal disease
PD11	Polycystic kidney disease
PD11z	Polycystic kidney disease NOS
PD11z-1	Cystic kidney disease NEC
PD13	Multicystic renal dysplasia
PD1y	Other specified congenital cystic kidney disease
PD1y0	Fibrocystic kidney disease
PD1y0-1	Fibrocystic renal degeneration
PD1yz	Other congenital cystic kidney disease NOS
PD1z	Congenital cystic kidney disease NOS
Pyu70	[X]Other cystic kidney diseases
Q48y0	Congenital renal failure
SK08	Acute renal failure due to rhabdomyolysis
SP154	Renal failure as a complication of care

SP154-1	Kidney failure as a complication of care
SP154-2	Post operative renal failure

10) Statins

ATCH52120NEMIS	Atorvastatin Chewable Tablets Sugar Free 10 mg
ATCH52123NEMIS	Atorvastatin Chewable Tablets Sugar Free 20 mg
ATOR21782NEMIS	Atorvastatin Oral suspension 20 mg/5 ml
ATOR45702NEMIS	Atorvastatin Oral suspension 10 mg/5 ml
ATOR45703NEMIS	Atorvastatin Oral solution 10 mg/5 ml
ATOR46710NEMIS	Atorvastatin Oral solution 20 mg/5 ml
ATTA30130EMIS	Atorvastatin Tablets 10 mg
ATTA30131EMIS	Atorvastatin Tablets 20 mg
ATTA30132EMIS	Atorvastatin Tablets 40 mg
ATTA6253NEMIS	Atorvastatin Tablets 80 mg
ATTA73968NEMIS	Atorvastatin Tablets 30 mg
ATTA73969NEMIS	Atorvastatin Tablets 60 mg
CATA41300NEMIS	Caduet Tablets 5 mg + 10 mg
CHTA111761NEMIS	Cholib Tablets 145 mg + 20 mg
CHTA111762NEMIS	Cholib Tablets 145 mg + 40 mg
CRTA15074NEMIS	Crestor Tablets 10 mg
CRTA15075NEMIS	Crestor Tablets 20 mg
CRTA15076NEMIS	Crestor Tablets 40 mg
CRTA21336NEMIS	Crestor Tablets 5 mg
DOM/43668NEMIS	Dorisin Xl M/R tablets 80 mg
EZTA20280NEMIS	Ezetimibe And Simvastatin Tablets 10 mg + 20 mg
EZTA20281NEMIS	Ezetimibe And Simvastatin Tablets 10 mg + 40 mg
EZTA20282NEMIS	Ezetimibe And Simvastatin Tablets 10 mg + 80 mg
FETA111758NEMIS	Fenofibrate And Simvastatin Tablets 145 mg + 20 mg
FETA111759NEMIS	Fenofibrate And Simvastatin Tablets 145 mg + 40 mg
FLCA24125EMIS	Fluvastatin Capsules 20 mg
FLCA24126EMIS	Fluvastatin Capsules 40 mg
FLTA5289NEMIS	Fluvastatin M/R tablets 80 mg
INTA20284NEMIS	Inegy Tablets 10 mg/20 mg
INTA20285NEMIS	Inegy Tablets 10 mg/40 mg
INTA20286NEMIS	Inegy Tablets 10 mg/80 mg
LECA24121EMIS	Lescol Capsules 20 mg
LECA24122EMIS	Lescol Capsules 40 mg
LETA5290NEMIS	Lescol Xl M/R tablets 80 mg
LICH52125NEMIS	Lipitor Chewable tablets 10 mg
LICH52126NEMIS	Lipitor Chewable tablets 20 mg
LITA10719BRIDL	Lipostat Tablets 10 mg
LITA10721BRIDL	Lipostat Tablets 20 mg
LITA1910NEMIS	Lipobay Tablets 400 micrograms
LITA30124EMIS	Lipitor Tablets 10 mg

LITA30125EMIS	Lipitor Tablets 20 mg
LITA30126EMIS	Lipitor Tablets 40 mg
LITA30703EMIS	Lipostat Tablets 40 mg
LITA30759EMIS	Lipobay Tablets 100 micrograms
LITA30760EMIS	Lipobay Tablets 200 micrograms
LITA30761EMIS	Lipobay Tablets 300 micrograms
LITA6254NEMIS	Lipitor Tablets 80 mg
LITA6646NEMIS	Lipobay Tablets 800 micrograms
LUM/35734NEMIS	Luvinsta Xl M/R tablets 80 mg
NAM/86955NEMIS	Nandovar Xl M/R tablets 80 mg
PIM/42037NEMIS	Pinmactil M/R tablets 80 mg
PROR105037NEMIS	Pravastatin Oral suspension 5 mg/5 ml
PROR43659NEMIS	Pravastatin Oral suspension 40 mg/5 ml
PROR81506NEMIS	Pravastatin Oral solution 5 mg/5 ml
PRTA10455HILLI	Pravastatin Tablets 10 mg
PRTA10456HILLI	Pravastatin Tablets 20 mg
PRTA30705EMIS	Pravastatin Tablets 40 mg
RATA18271NEMIS	Ranzolont Tablets 10 mg
RATA18272NEMIS	Ranzolont Tablets 20 mg
RATA18273NEMIS	Ranzolont Tablets 40 mg
ROTA15069NEMIS	Rosuvastatin Tablets 10 mg
ROTA15071NEMIS	Rosuvastatin Tablets 20 mg
ROTA15072NEMIS	Rosuvastatin Tablets 40 mg
ROTA21335NEMIS	Rosuvastatin Tablets 5 mg
SIOR20422NEMIS	Simvastatin Oral suspension 20 mg/5 ml
SIOR25476NEMIS	Simvastatin Oral suspension 40 mg/5 ml
SIOR44785NEMIS	Simvastatin Oral Suspension (Sugar-Free) 20 mg/5 ml
SIOR44786NEMIS	Simvastatin Oral Suspension (Sugar-Free) 40 mg/5 ml
SIOR45597NEMIS	Simvastatin Oral solution 20 mg/5 ml
SIOR45598NEMIS	Simvastatin Oral solution 40 mg/5 ml
SITA10076BRIDL	Simvastatin Tablets 10 mg
SITA10078BRIDL	Simvastatin Tablets 20 mg
SITA16195NEMIS	Simvador Tablets 10 mg
SITA16196NEMIS	Simvador Tablets 20 mg
SITA16197NEMIS	Simvador Tablets 40 mg
SITA29406EMIS	Simvastatin Tablets 40 mg
SITA34996NEMIS	Simvador Tablets 80 mg
SITA3663NEMIS	Simvastatin Tablets 80 mg
STM/44675NEMIS	Stefluvin Xl M/R tablets 80 mg
ZOTA29404EMIS	Zocor Tablets 40 mg
ZOTA3664NEMIS	Zocor Tablets 80 mg
ZOTA8622EGTON	Zocor Tablets 10 mg
ZOTA8623EGTON	Zocor Tablets 20 mg

11) Fibrates

ATCA239	Atromid-S Capsules 500 mg
BEM/34165EMIS	Bezagen Xl M/R tablets 400 mg
BETA362	Bezalip Tablets 200 mg
BETA363	Bezalip-Mono M/R tablets 400 mg
BETA4655	Bezafibrate Tablets 200 mg
BETA4656	Bezafibrate M/R tablets 400 mg
CITA21919EMIS	Ciprofibrate Tablets 100 mg
CLCA647	Clofibrate Capsules 500 mg
FECA1203NEMIS	Fenofibrate Capsules (Micronised) 267 mg
FECA13841NEMIS	Fenogal Capsules 200 mg
FECA18893NEMIS	Fenofibrate Capsules (Micronised) 200 mg
FECA24144EMIS	Fenofibrate Capsules 200 mg
FECA31990EMIS	Fenofibrate Capsules (Micronised) 67 mg
FECA9492HILLI	Fenofibrate Capsules 100 mg
FETA5643NEMIS	Fenofibrate Tablets (Micronised) 160 mg
FIM/51928NEMIS	Fibrazate Xl M/R tablets 400 mg
LICA1205NEMIS	Lipantil Micro 267 Capsules 267 mg
LICA1207NEMIS	Lipantil Micro 200 Capsules 200 mg
LICA22524NEMIS	Lipanthyl 300 Capsules
LICA24142EMIS	Lipantil Micro Capsules 200 mg
LICA9431BRIDL	Lipantil Capsules 100 mg
LIM/3539NEMIS	Liparol Xl M/R tablets 400 mg
LITA31988EMIS	Lipantil Micro 67 Capsules 67 mg
MOTA21917EMIS	Modalim Tablets 100 mg
SUTA5645NEMIS	Supralip Tablets 160 mg
ZIM/5338NEMIS	Zimbacol Xl M/R tablets 400 mg

12) Bile Acid Sequestrants

CHPO612	Cholestyramine Powder 4g
CHSU15277NEMIS	Cholestyramine Sugar Free Powder 4 grams/sachet
CHTA27652NEMIS	Cholestagel Tablets 625 mg
COGR4776	Colestipol Hydrochloride Sugar free granules 5 grams/sachet
COGR713	Colestid Granules Plain, 5 grams/sachet
COOR17597NEMIS	Colestyramine Oral Powder (sachets) 4 grams/sachet
COOR17598NEMIS	Colestyramine Oral Powder Sachets Sugar Free 4 grams/sachet
COSA21955EMIS	Colestid Orange Sachets 5 grams/sachet
COSA21957EMIS	Colestipol Hydrochloride Sachets (orange) 5 grams/sachet
MUTA3984	Muripsin Tablets
QUPO2421	Questran Powder 4g
QUPO9528BRIDL	Questran Light Powder 4 grams/sachet

13) Nicotinic Acid

BRTA395	Bradilan Tablets 250 mg

BRTA396	Bradilan Tablets 500 mg
NACA48437NEMIS	Nature's Bounty Time Released Niacin Capsules 250 mg
NIM/14469NEMIS	Nicotinic Acid M/R tablets 500 mg
NIM/16456NEMIS	Nicotinic Acid M/R tablets 750 mg
NIM/16457NEMIS	Nicotinic Acid M/R tablets 1 gram
NIM/16459NEMIS	Niaspan M/R tablets 500 mg
NIM/16460NEMIS	Niaspan M/R tablets 750 mg
NIM/16461NEMIS	Niaspan M/R tablets 1 gram
NIM/16463NEMIS	Nicotinic Acid Titration Starter Pack M/R tablets 7 x 375 mg, 7 x 500 mg, 7 x 750 mg
NIM/16465NEMIS	Niaspan Titration Starter Pack M/R tablets 7 x 375 mg, 7 x 500 mg, 7 x 750 mg
NIM/33335NEMIS	Nicotinic Acid And Laropiprant M/R tablets 1 gram + 20 mg
NITA1983	Nicotinic Acid Tablets 100 mg
NITA1984	Nicotinic Acid Tablets 25 mg
NITA1985	Nicotinic Acid Tablets 50 mg
NITA5202	Nicofuranose Tablets 250 mg
TRM/38503NEMIS	Tredaptive M/R tablets 1 gram + 20 mg

14) PCSK9 Inhibitors

EVSO105025NEMIS	Evolocumab Solution for injection 140 mg/1 ml pre-filled syringe
EVSO105029NEMIS	Evolocumab Solution for injection 140 mg/1 ml pre-filled device
RESO105028NEMIS	Repatha Solution for injection 140 mg/1 ml pre-filled syringe
RESO105031NEMIS	Repatha Sureclick Solution for injection 140 mg/1 ml pre-filled device
